# The Potential Contribution of the Scientific Diaspora to Enhance Marine Science in Guatemala

**DOI:** 10.3389/frma.2022.898082

**Published:** 2022-06-13

**Authors:** Carmen Barrios-Guzmán, Diego de la Cruz

**Affiliations:** ^1^Laboratorio de Ecología de Mamíferos Marinos (LECMMAR), Instituto Biología, Facultad de Ciencias, Universidad de Valparaíso, Valparaíso, Chile; ^2^Independent Researcher, Guatemala City, Guatemala

**Keywords:** Guatemala, international cooperation, knowledge transfer, Latin America, marine science, science cooperation, scientific diaspora, scientific networks

## Introduction

The republic of Guatemala has 402 km of coastline, including the Caribbean Sea and the Eastern Pacific Ocean (CONAP, 2009). Despite having a privileged geographic location and rich marine-coastal biodiversity, it has been considered that “*Guatemala has lived with its back to the sea”* (Carrera et al., 2012; González-Bernat and Clifton, 2017). This fact is observed through a substantial lack of data and information, which denotes that those marine resources in Guatemala are understudied and subsequently poorly managed. Partially because of poor inter-institutional coordination, scant budget allocation, and lack of human resources (Carrera et al., 2012; González-Bernat and Clifton, 2017, 2021a,b; Caviedes et al., 2021). One of the most relevant reasons that lead to poorly marine resources management is the lag in science and technology in the country.

Limited offer of university programs is insufficient both in terms of coverage (number of programs available) and quality (part-time dedication with little scientific production) (Bonilla, 2021; RICYT, 2021; Martínez, 2022). Consequently, for decades some Guatemalans tend to apply for international cooperation scholarships, university discounts, student loans, or their own sponsorship to educate themselves (Bonilla and Kwak, 2015; Bonilla, 2021). Once graduated, these professionals often decide to establish their residence and workplace in other countries, among other factors, due to the lack of job opportunities and professional development in the universities of their home country (Charum and Meyer, 1998; Mera, 2011; Bonilla, 2021). Therefore, these Guatemalan scientists residing abroad make up the Guatemalan scientific diaspora.

The scientific diaspora could become a resource of interest for the country since it is a group of qualified people who know well the culture of the country where they reside. It might be the perfect opportunity to connect with academic and scientific actors from the productive sectors and government entities (Echeverría-King and Prieto, 2021). Also, they could be a bridge for the execution of cooperation projects and activities, facilitating the exchange and transfer of knowledge and technology (Palacios-Callender and Roberts, 2018; Echeverría-King and Prieto, 2021; Lopez-Verges et al., 2021). In this context, this article aims to describe the potential of the scientific diaspora and how it would contribute to strengthening this area of knowledge.

## Science in Guatemala

The latest report on the State of Science (2021) places Guatemala as one of the countries with low scientific development and a possible cause is the scant budget allocation and lack of human resources. Guatemala barely invests 0.03% of the gross domestic product of science and technology. The concern is that this allocation has not changed in the last decade due to the government and private companies' lack of interest and commitment to granting the necessary economic resource to the development of science and research (Martínez, 2022). In 2019, the spending on investment and development in the country was US$39.81 million (including the investment in the academic sector and the state; RICYT, 2021). Therefore, Guatemala continues to be a country that does not invest in science since it does not consider this a priority. This fact occurs because it is not understood that the material wealth of countries goes hand in hand with their technological development and the fact that investment in science is a long-term strategy that supports the management of biological resources (Pazos, 2020).

Furthermore, the number of researchers in the country is the lowest in the Central American region and Latin America. Guatemala reported 508 researchers (2019), while Costa Rica and El Salvador reported 3,781 (2018), and 1,030 (2019) researchers, respectively. Meanwhile, the benchmark of South America is Argentina and Brazil, with 90,747 (2019) and 421,838 (2018) researchers, respectively. Therefore, this places Guatemala as the country with the lowest number of researchers and limited scientific production. In 2019 Guatemala registered in SCOPUS 2.2 publications per 100.000 inhabitants, while in the same year, Costa Rica, Argentina, and Brazil registered 27.2, 33.2, and 41.6 publications, respectively (RICYT, 2021).

The positive trend for national scientific production in Guatemala in the last decade has been increasing, with 147 publications in 2010, 232 in 2013, 283 in 2016, and 357 in 2019 (Monge-Nájera and Ho, 2018; RICYT, 2021). This positive trend is also indicated in the latest report on the State of Science (2021), which report that between 2016 and 2019, Guatemala dedicated 53.3% of its scientific production to issues related to one of the Sustainable Development Goals (RICYT, 2021). This is encouraging, knowing that the scientific community has shown a greater interest in topics that aim to study these goals that define all the priorities that exist at the global level concerning the significant challenges that are faced to advance sustainable development (UNDP, 2022).

## Marine Science and Guatemalan Scientific Diaspora

After agreeing with Carrera et al. (2012) and González-Bernat and Clifton (2017) that “*Guatemala has lived with its back to the sea,”* it is necessary to establish the reasons why the development of marine science in Guatemala has not been given the relevance it deserves. At least two factors could explain these causes:

- *Limited offer of university programs*: Most of these professionals have received their training in two main ways. (1) Pursue an undergraduate degree in Biology, or undergraduate degree in Hydrobiological Resources and Aquaculture that eventually allows specialization in marine sciences, (2) Pursue a postgraduate degree in Guatemala (master's degree in Marine and Coastal Sciences is the only option), or abroad (MSc or Ph.D. in Marine Science, Oceanography or related). Thus, many of these professionals do not have training in marine sciences *per se* but rather become trained in this area of knowledge at a later stage of their degree.
- *Low and Insufficient Funding*: In 2019, Latin America made an investment that represented 0.56% of the gross domestic product of science and technology. The Central America region invests between 0.03 and 0.39%, while the South America region invests between 0.14 and 1.6% (RICYT, 2021).

Although both factors result in a shortage of skilled scientists and technical workforce to cover the country's needs for research & development (Tarifeño-Silva, 2002), in the last decade, a positive trend in national scientific production prevails in Guatemala, which is an encouraging step for the country development. In marine sciences, it is not the exception. However, beyond the evident increase in scientific activity in marine science in Guatemala, the degree of development does not seem sufficient to address the main emerging issues of the discipline, which was recently outlined in the international agenda (National Research Council (NRC)., 2015; Intergovernmental Oceanographic Commission - United Nations Educational, 2017). Most of the scientific publications related to this area of knowledge in the last decade, agree that one of the main problems in Guatemala is the lack of data and updated information. Such as, the description of the fishing fleet and its landings, consumption of hydrobiological products and their production, and the distribution and abundance of marine species, among others (e.g., Brittain, 2016; González-Bernat and Clifton, 2017, 2021a,b; Hernández-Padilla et al., 2020; Muñoz et al., 2021).

The Guatemalan higher education system does not offer the conditions to train and educate marine science scientists and lacks permanent financing for adequate scientific development. This led to the notion that there are several highly-trained professionals in marine sciences in Guatemala's scientific diaspora. To try to identify and characterize this scientific diaspora, the directory of members of four organizations that bring together Guatemalan professionals who do science in the country or abroad was consulted. The total members of the organizations and the number of professionals working in marine sciences were recorded, also identifying whether they work abroad (Table 1).

**Table 1:** Number of marine sciences professionals and researchers that are registered in a Science and Technology directory in Guatemala.

**Organization**	**Total Members**	**Marine Science Members**	**Scientific Diaspora^**(a)**^**
International Network of Science, Technology, and Innovation (RedCTI)^1^	194	1	1
Academy of Medical, Physical and Natural Sciences^2^	86	-	-
National Directory of Researchers of the National Secretariat of Science and	16^†^	6*	1
Technology (DIN- SENACYT)^3^
Organization of Women in Science for the Developing	440	12*	6
World–Guatemala chapter (OWSD-GT)^4^

*Academy of Sciences–Directory of members is restricted in three general areas, Physical, Natural and Medical Sciences*.

DIN-SENACYT–In the directory it is not possible to search by specific profession. For this, the research catalog of the area of Earth, Ocean and Space Sciences was reviewed. (*) Includes only ocean science research.

*OWSD-GT–Members that indicate their relationship with marine sciences among their academic background, research, and profession were reviewed.

*aScientific Diaspora–The same professional registered in three organizations*.

^1^*RedCTI https://redcti.senacyt.gob.gt/portal/index.php*.

^2^*Academy of Medical, Physical and Natural Sciences https://www.acacienciasgt.org/index.php/2-uncategorised/8-people*.

^3^*National Directory of Researchers of the National Secretariat of Science and Technology https://fondo.senacyt.gob.gt/portal/index.php/catalogo*.

^4^*OWSD Guatemala https://owsd.net/network/guatemala*.

Nineteen marine science professionals registered in one or more of these organizations have been identified. However, most of the members are registered under a university's affiliation without specifying a faculty or research center. They also do not indicate academic background, research, and specific profession. Therefore, it was impossible to identify several of these members if they worked in this area of knowledge. Also, through a systematic search of available literature related to marine sciences in Guatemala in the ISI Web of Science and SCOPUS databases, it was found that some of these authors are not registered in any of these organizations, and some have affiliations with the prominent universities of the country. This agrees with Monge-Nájera and Ho, (2018) that the authors with the most significant scientific production in Guatemala coincide with the leading institutions and suggests that high-quality research depends, to a large extent, on individual researchers who lead production in institutions.

The initial search identified 19 marine science professionals, of which 6 are part of the scientific diaspora (although one is registered in three of the four organizations consulted; Table 1). Of these, only three contributed to the scientific development of marine science in Guatemala, with scientific publications in the last decade. Perhaps more professionals have contributed to scientific production or knowledge transfer and cooperation, but we do not know because much of the work done by diasporas is not published and is therefore under-reported. Also, these organizations that bring together Guatemalan scientists and professionals must periodically update their database, and this information must be more dynamic and accessible, so that the benefits are seen as more participatory.

## Linking the Scientific Diaspora

Regardless of location, it has been described that the scientific diaspora can actively contribute to (1) *Strengthen the higher education system*, contributing to the design of national and regional postgraduate programs, and increasing the offer of these university programs, (2) *Increase productivity and scientific impact*, (3) *Generate mobility opportunities* (executing projects, cooperation activities, facilitate the exchange, and transfer of knowledge and technology), and (4) *Be a bridge between science and decision makers*, guiding government policies and regulations (Scientific Diplomacy) (ICMPD, International Centre for Migration Policy Development, 2019; Bonilla, 2021; Lopez-Verges et al., 2021). Also, a well-connected diaspora may aid reinsertion strategies (Stehli, 2020) and help design national and regional postgraduate programs that could increase intraregional mobility, strengthen regional collaboration, and increase productivity and visibility of research (Lopez-Verges et al., 2021).

The linkage mechanisms could start with the joint participation of the Ministry of Foreign Affairs with the National Secretariat of Science and Technology (and other relevant actors, e.g., the National Academy of Sciences, Universities) to identify this scientific diaspora and generate dialogue between several actors involved. Also, to map the scientific diaspora, a website can be created through several initiatives that allows the registration of these scientists abroad to understand how many, where they are, and the paths of these scientists around the world. At this same line, two successful cases can be mentioned of mapping the scientific diaspora, Portugal (GPS, 2022) and Costa Rica (Marques et al., 2020; HIPATIA, 2022; Pasamontes, 2022). The global health and economic crisis caused by the COVID19 pandemic promoted higher informal networking through social networks (Twitter, Facebook, Instagram). This served, and could continue, as a tool to identify, connect and create conversation spaces with some members of the diaspora.

## Discussion

Undoubtedly, one of the greatest challenges that is facing not only Guatemala, but also several countries in the region and the world, is the national investment in science, technology, and innovation. Guatemala barely invests 0.03% of the gross domestic product (equivalent to US$ 2.40 per inhabitant), which means that a large part of the advanced human capital leaves or remains outside the country, with the subsequent costs for the development of sciences in Guatemala. For this reason, it is necessary to improve dialogue and coordination between the sectors for the development of joint actions that allow opening spaces for communication with the scientific diaspora for the generation of alliances and cooperation that, from the scientific perspective, have an impact on social benefit.

Due to the lack of research in this field in Guatemala, it is hoped to have made a helpful initial contribution and have highlighted some of the core aspects of the contribution of the scientific diaspora. Given the urgency of the current challenges facing the oceans, all available methods to support effective and equitable responses to your study should be used to the best of their ability. It is believed that the link with the diaspora can be important in this matter, by strengthening the system of science and higher education both in Guatemala and in the region.

Finally, it is suggested as a good start, to map the marine scientific diaspora through a systematic and quantitative review of the publications of Guatemalan authors and to characterize their international collaborative networks.

## Author Contributions

Both authors have made a substantial, direct, and intellectual contribution to the work and approved it for publication.

## Conflict of Interest

The authors declare that the research was conducted in the absence of any commercial or financial relationships that could be construed as a potential conflict of interest.

## Publisher's Note

All claims expressed in this article are solely those of the authors and do not necessarily represent those of their affiliated organizations, or those of the publisher, the editors and the reviewers. Any product that may be evaluated in this article, or claim that may be made by its manufacturer, is not guaranteed or endorsed by the publisher.

## References

[B1] Bonilla K. (2021). La diáspora científica centroamericana: Oportunidades de vinculación y fortalecimiento de las universidades y la educación superior en el istmo. Revista de Educación Superior en América Latina. Universidad del Norte. Barranquilla, Colombia. Available online at: https://www.uninorte.edu.co/web/revista-esal/inicio (accessed May 8, 2022).

[B2] Bonilla K. Kwak J. S. (2015). Effectiveness of donor support for capacity development in Guatemala: a study of scholarship provision for overseas postgraduate education. Iberoamerica 17, 293–344.

[B3] Brittain R. (2016). Assessing interactions between marine megavertebrates and small-scale fisheries on the Pacific coast of Guatemala (Master's thesis). Cornwall, UK: University of Exeter.

[B4] Carrera J. L. López-Selva M. M. López E. (2012). Zona marino-costera: nuevas olas extractivas (Perfil Ambiental de Guatemala 2010–2012. Vulnerabilidad local y creciente construcción de riesgo), Universidad Rafael Landí*var, a través del Instituto de Agricultura, Recursos Naturales y Ambiente (IARNA) de Guatemala*. Centroamérica, Guatemala. 147–160.

[B5] Caviedes V. Arenas-Granados P. Barragán-Muñoz J. M. (2021). Progress for integrated coastal zone management in the Caribbean of Guatemala. Rev. Cien. Amb. 55, 254–275. 10.15359/rca.55-2.13

[B6] Charum J. Meyer J. B. (1998). ¿*El nuevo nomadismo cient*í*fico? La perspectiva latinoamericana*. Bogotá, Escuela Superior de Administración Pública.

[B7] CONAP M. (2009). Biodiversidad Marina de Guatemala: Análisis de vacíos y estrategias para su conservación, Consejo Nacional de Áreas Protegidas, Ministerio de Ambiente y Recursos Naturales. Guatemala: The Nature Conservancy.

[B8] Echeverría-King L. F. Prieto J. J. (2021). Diáspora científica en el Sur Global:? por qué es importante para Colombia? Coordenadas Mundiales. El blog de la Escuela de Relaciones Internacionales de FIGRI. Universidad Externado de Colombia. Bogotá, Colombia. Available online at: https://coordenadas-mundiales.uexternado.edu.co/diaspora-cientifica-en-el-sur-global-por-que-es-importante-para-colombia/ (accessed January 8, 2022).

[B9] González-Bernat M. J. Clifton J. (2017). “Living with our backs to the sea”: a critical analysis of marine and coastal governance in Guatemala. Mar. Policy, 81:9–20. 10.1016/j.marpol.2017.03.003

[B10] González-Bernat M. J. Clifton J. (2021a). A governance analysis of two marine protected areas in the Pacific Region of Guatemala: the multiple use area of Monterrico and the Private Reserve La Chorrera-Manchón Guamuchal. Mar. Policy 127:103625. 10.1016/j.marpol.2019.103625

[B11] González-Bernat M. J. Clifton J. (2021b). A governance analysis of Guatemala's first recognized marine protected area: The Wildlife Refuge of Punta de Manabique (RVSPM). Mar. Policy, 127, 103626. 10.1016/j.marpol.2019.103626

[B12] GPS. (2022). Global Portuguese Scientists. Available online at: https://gps.pt/ (accessed May 13, 2022).

[B13] Hernández-Padilla J. C. Capetillo-Piñar N. Vélez-Arellano N. Aranceta-Garza F. Ortíz-Aldana J. R. Navas-Beteta A.. (2020). Variación espacial en la composición y abundancia de las especies capturadas por las pesquerías de pequeña escala en el litoral del Pacífico de Guatemala. Revista Mesoamericana de Biodiversidad y Cambio Climático–Yu'am 4, 19–43. Available online at: https://www.revistayuam.com/variacion-espacial-en-la-composicion-y-abundancia-de-las-especies-capturadas-por-las-pesquerias-de-pequena-escala-en-el-litoral-del-pacifico-de-guatemala/

[B14] HIPATIA. (2022). Portal HIPATIA: Estado de las capacidades en Ciencia, Tecnología e Innovación. Available online at: https://hipatia.cr (accessed May 13, 2022).

[B15] ICMPD International Centre for Migration Policy Development. (2019). Empowering diaspora to be the future bridges of investment, education, and Innovation. Observations and Recommendations from Diaspora Studies in Germany, and Ireland Best Practices from EU Member States. Migration Governance Seminar - 10 July 2019. Available online at: https://www.ilo.org/wcmsp5/groups/public/---asia/---ro-bangkok/---sro-new_delhi/documents/presentation/wcms_717577.pdf (accessed January 6, 2022).

[B16] Intergovernmental Oceanographic Commission - United Nations Educational Scientific, and Cultural Organization (IOC-UNESCO). (2017). Global Ocean Science Report. The current status of ocean science around the world. Paris: UNESCO Publishing.

[B17] Lopez-Verges S. Valiente-Echeverría F. Godoy-Faúndez A. Rivas D. F. Urbani B. Berger J. J.. (2021). Call to action: Supporting Latin American early career researchers on the quest for sustainable development in the region. Front. Res. Metr. Anal. 6, 657120. 10.3389/frma.2021.657120 34056515 PMC8160458

[B18] Marques J. L. Bressan G. Santos C. Pedro L. Marçal D. Junior E.. (2020). “Global Portuguese Scientists (GPS): an academic social network to assess mobility in science,” in ICDSST 2020 Proceedings-Online Version the EWGDSS 2020 International Conference on Decision Support System Technology I (Zaragoza), 235–241.

[B19] Martínez B. (2022). Rezago en ciencia e investigación persiste en el paí*s*. Guatemala: Prensa Libre, Guatemala. Available online at: https://www.prensalibre.com/pl-plus/guatemala/comunitario/rezago-en-ciencia-e-investigacion-persiste-en-guatemala/

[B20] Mera C. (2011). “Comparación de las diásporas en Asia y América Latina como factores de desarrollo,” sCirculación de saberes y movilidades internacionales: perspectivas latinoamericanas, eds Hernández, V. et al. (coords.), (Buenos Aires:Biblos).

[B21] Monge-Nájera J. Ho Y. S. (2018). Guatemala articles in the Science Citation Index Expanded: bibliometry of subjects, collaboration, institutions, and authors. Rev. Biol. Trop., 66, 312–320. 10.15517/rbt.v66i1.29875

[B22] Muñoz A. de la Cruz D. Josupeit H. (2021). Cadena de valor de los productos de la pesca artesanal en Guatemala. Circular de Pesca y Acuicultura de la FAO No. 1202. Roma, FAO.

[B23] National Research Council (NRC). (2015). Seachange: 2015-2025. Decadal Survey of Ocean Sciences. Washington, DC: National Academies Press.

[B24] Palacios-Callender M. Roberts S. A. (2018). Scientific collaboration of Cuban researchers working in Europe: understanding relations between origin and destination countries. Scientometrics 117, 745–769. 10.1007/s11192-018-2888-2 30595611 PMC6280978

[B25] Pasamontes M. S. (2022). Diáspora científica costarricense: un valioso activo para vincular. Revista de Educación Superior en América Latina. Universidad del Norte. Barranquilla, Colombia. Available online at: https://rcientificas.uninorte.edu.co/index.php/esal/article/view/14231

[B26] Pazos E. (2020). Somos pobres porque no invertimos en ciencia y tecnología. Guatemala: Prensa Libre. Available online at: https://www.prensalibre.com/vida/tecnologia/somos-pobres-porque-no-invertimos-en-ciencia-y-tecnologia/ (accessed February 6, 2022).

[B27] RICYT. (2021). Red de Indicadores de Ciencia y Tecnología -Iberoamericana e Interamericana. EL ESTADO DE LA CIENCIA. Principales Indicadores de Ciencia y Tecnología Iberoamericanos/Interamericanos 2021. Ciudad Autónoma de Buenos Aires, Argentina. Available online at: http://www.ricyt.org/2021/11/ya-se-encuentra-disponible-el-estado-de-la-ciencia-2021/ (accessed May 13, 2022).

[B28] Stehli M. (2020). Dossier completo Migración calificada y Movilidad académica en América Latina, tendencias, perspectivas y nuevas líneas de investigación. Formarse y volver. Controv. Concurr. Lat. 12, 107–135. Available online at: https://www.researchgate.net/publication/347928883_Dossier_completo_Migracion_calificada_y_Movilidad_academica_en_America_Latina_Debates_tendencias_perspectivas_y_nuevas_lineas_de_investigacion

[B29] Tarifeño-Silva E. (2002). North–South educational partnership on marine sciences: the Latin American experiences and perspectives. Ocean Coast Manag. 45:649–666. 10.1016/S0964-5691(02)00091-1

[B30] UNDP. (2022). United Nations Development Programme. The SDGS in action. Sustainable Development Goals. Available online at: https://www.undp.org/sustainable-development-goals?utm_source=EN&utm_medium=GSR&utm_content=US_UNDP_PaidSearch_Brand_English&utm_campaign=CENTRAL&c_src=CENTRAL&c_src2=GSR&gclid=EAIaIQobChMIpLKTrpCt9wIVROZcCh2CawM_EAAYAyAAEgLEe_D_BwE (accessed April 13, 2022).

